# Conformational dynamics of the HIV-1 envelope glycoprotein from CRF01_AE is associated with susceptibility to antibody-dependent cellular cytotoxicity

**DOI:** 10.1128/jvi.01667-25

**Published:** 2025-12-09

**Authors:** Marco A. Díaz-Salinas, Mehdi Benlarbi, Debashree Chatterjee, Manon Nayrac, Megane Robidas, Suteeraporn Pinyakorn, Nittiya Phanuphak, Carlo Sacdalan, Halima Medjahed, Jérémie Prévost, Lydie Trautmann, Marzena Pazgier, Andrés Finzi, James B. Munro

**Affiliations:** 1 Department of Microbiology, University of Massachusetts Chan Medical School 12262, Worcester, Massachusetts, USA; 2 Centre de Recherche du CHUM, Montréal, Québec, Canada; 3 Département de Microbiologie, Infectiologie et Immunologie, Université de Montréal 5622, Montréal, Québec, Canada; 4 U.S. Military HIV Research Program, Walter Reed Army Institute of Research 8394, Silver Spring, Maryland, USA; 5 Henry M. Jackson Foundation for the Advancement of Military Medicine, Inc., Bethesda, Maryland, USA; 6 SEARCH, Institute of HIV Research and Innovation 606508, Bangkok, Thailand; 7 Faculty of Medicine, Chulalongkorn University 26683, Bangkok, Thailand; 8 Vaccine and Gene Therapy Institute, Oregon Health & Science University, Beaverton, Oregon, USA; 9 Infectious Diseases Division, Department of Medicine, Uniformed Services University of the Health Sciences 1685, Bethesda, Maryland, USA; 10 Department of Biochemistry and Molecular Biotechnology, University of Massachusetts Chan Medical School 12262, Worcester, Massachusetts, USA; The Ohio State University, Columbus, Ohio, USA

**Keywords:** smFRET, HIV, Env, CRF01_AE, ADCC

## Abstract

The HIV-1 envelope glycoprotein (Env) is expressed at the surface of infected cells and, as such, can be targeted by non-neutralizing antibodies (nnAbs) that mediate antibody-dependent cellular cytotoxicity (ADCC). Previous single-molecule Förster resonance energy transfer (smFRET) studies demonstrated that Envs from clinical isolates predominantly adopt a “closed” conformation (State 1), which is resistant to nnAbs. After interacting with the cellular receptor CD4, the conformational equilibrium of Env shifts toward States 2 and 3, exposing the coreceptor-binding site (CoRBS) and permitting targeting by CD4-induced (CD4i) antibodies. We showed that the binding of anti-CoRBS Abs enables the engagement of other nnAbs that target the cluster A epitopes on Env. Anti-cluster A nnAbs stabilize an asymmetric Env conformation, State 2A, and have potent ADCC activity. CRF01_AE strains were suggested to be intrinsically susceptible to ADCC mediated by nnAbs. This may be due to the presence of a histidine at position 375, known to shift Env toward more “open” conformations. In this work, through adaptation of an established smFRET imaging approach, we report that native, unliganded CRF01_AE HIV-1 Envs frequently sample the State 2A conformation. This is in striking contrast with Envs from clades A and B, for example HIV-1_JR-FL_, which do not transition to State 2A in the absence of ligands. These findings inform on the conformational dynamics of CRF01_AE Env, which are relevant for structure-based design of both synthetic inhibitors of receptor binding and enhancers of ADCC as therapeutic alternatives.

**IMPORTANCE:**

A concerning increase in infections with HIV-1 from CRF01_AE has occurred globally and regionally in recent years, especially in Southeast Asia. Despite the advances made in understanding HIV-1 envelope glycoprotein (Env) conformational dynamics, the knowledge about Env from CRF01_AE HIV-1 is limited. Here, we demonstrate that the unliganded CRF01_AE Env readily samples an “open” conformation (State 2A), which is susceptible to antibody-dependent cellular cytotoxicity (ADCC). This is in contrast with the subtypes previously studied from HIV-1 group M that rely on anti-cluster A antibodies to adopt State 2A. These findings are relevant for the structure-based design of novel synthetic inhibitors of CD4 binding and enhancers of ADCC for the elimination of infected cells.

## INTRODUCTION

The RV144 HIV-1 vaccine trial in Thailand, which concluded in 2009, elicited a 31.2% protective efficacy. Subsequent analyses indicated that this modest protection was correlated with antibodies (Abs) with Ab-dependent cellular cytotoxicity (ADCC) activity specific to the HIV-1 envelope glycoprotein (Env) in a subset of individuals with low plasma IgA (1, 2). This suggests that ADCC may have contributed to the protection observed in the RV144 trial. HIV-1 strains of the circulating recombinant form AE (CRF01_AE) predominate the AIDS epidemic in Southeast Asia (3, 4). Therefore, the RV144 trial used an ALVAC-HIV prime and AIDSVAX B/E boost regimen, which included one CRF01_AE (A244) and one clade B (MN) gp120 glycoprotein. Moreover, the prevalence of HIV-1 CRFs has risen in recent years, most significantly in Southeast Asia (5). For these reasons, a detailed investigation of Env from HIV-1 CRFs is warranted. While advances in the understanding of Env conformational dynamics have been achieved using virological and biophysical approaches, these studies have focused on HIV-1 subtypes A and B (6–12). A similar elucidation of the dynamics of Env from HIV-1 CRFs has not been reported. However, prior studies demonstrated an inherent susceptibility of CRF01_AE HIV-1 to ADCC, which begins to explain the results of the RV144 trial (6, 13). Subsequent structural investigation of CRF01_AE Env indicated features that are distinct from other subtypes and perhaps enable conformations related to recognition by Abs with ADCC activity (14). In the present study, we explore the conformational features of Envs from CRF01_AE and their relationship to ADCC mediated by plasma from people living with HIV (PLWH).

The first step in the replication of HIV-1 is the binding of Env to the cellular receptor CD4. Env is synthesized as the gp160 precursor, which is trimerized and glycosylated in the endoplasmic reticulum of infected cells (15, 16), followed by proteolytic processing by host furin-like proteases in the Golgi apparatus (17–19). The resulting cleaved and mature Env trimer is composed of three gp120 subunits, which are non-covalently associated with three transmembrane gp41 subunits [(gp120-gp41)_3_] (20–22). Mature Env is present on virions as well as exposed on the surface of infected cells, making it the primary target of host Abs. Some Abs neutralize the virus (NAbs) by blocking Env’s interaction with receptors or inhibiting conformational changes needed to promote fusion of the viral and cellular membranes. Other Abs that are frequently elicited during HIV-1 infection, including in PLWH, are non-neutralizing (nnAbs) since they recognize Env epitopes occluded within “closed” Env conformations. Certain classes of nnAbs, however, can induce the death of infected cells through ADCC, provided Env samples an “open” conformation.

Single-molecule Förster resonance energy transfer (smFRET) imaging studies demonstrated that Env is highly dynamic, transitioning from a “closed” conformation (State 1) to an “open” conformation (State 3), which is promoted through the interaction with CD4. An asymmetric intermediate (State 2) of Env can be observed during the transition from State 1 to State 3 (10, 12). The Env conformational equilibrium from primary HIV-1 isolates of clades A and B favors State 1 in the absence of ligands, which confers resistance to most Abs, especially those that target CD4-induced (CD4i) epitopes (12, 23). Nonetheless, some broadly neutralizing Abs (bNAbs) preferentially bind this “closed” conformation (8, 12). However, after interacting with cellular CD4, Env adopts State 3, exposing cryptic epitopes including the coreceptor-binding site (CoRBS) and gp120 cluster A region, which can be targeted by nnAbs to promote ADCC (6, 23–28). CD4-mimetic compounds (CD4mcs) are small molecules designed to target specifically the CD4 binding cavity within HIV-1 Env. CD4mcs can induce conformational changes in Env that sensitize it to recognition by nnAbs (25, 26). In the presence of soluble CD4 (sCD4) or CD4mcs, anti-CoRBS and anti-cluster A Abs stabilize State 2A, which is an asymmetric Env conformation associated with increased ADCC responses *in vitro* and Fc-effector functions *in vivo* (9, 25, 29–31).

The findings presented here indicate that native HIV-1 Envs from tier-1 and tier-2 CRF01_AE strains intrinsically sample the State 2A conformation, which is susceptible to ADCC even in the absence of CD4 or CD4mcs. This contrasts with clade-B HIV-1_JR-FL_ Env, which depends on interaction with CD4 or CD4mcs and antibodies targeting the CoRBS to adopt State 2A (9, 25). Interaction of the tier-1 CRF01_AE Env with CD4 and CoRBS Abs further stabilized State 2A. The conformational features of CRF01_AE Envs warrant further research to identify the structural determinants or elements that govern its dynamic equilibrium shift to more “open” conformations. Targeting CRF01_AE HIV-1-infected cells with cocktails of antibodies (32) or molecules able to recognize downstream Env conformations represents promising strategies to decrease the reservoir of individuals living with this viral strain (33).

## RESULTS

### CRF01_AE HIV-1-infected cells are more susceptible to ADCC than a representative subtype B strain

**Fig 1:**
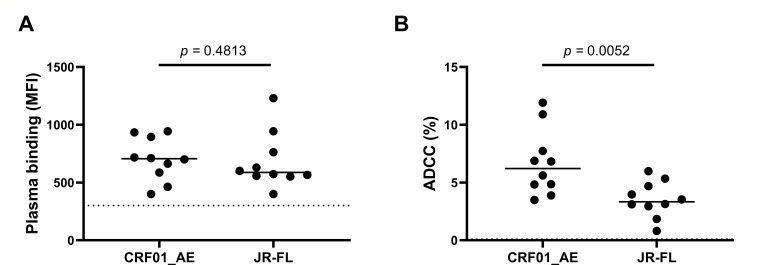
HIV-1_CRF01_AE_ strain 703357 is more susceptible to ADCC than HIV-1_JR-FL_. (**A**) Binding of plasma from PLWH to primary CD4+ T cells infected with the indicated HIV-1 strains was evaluated. Five independent experiments (*n* = 5) were performed with the mean of each one of the 10 plasma samples plotted as individual dots. Means are shown as horizontal bars. (**B**) ADCC responses to the indicated viral strains. Data are plotted as in panel **A**. In this case, the numbers of independent experiments were 5 and 4 for HIV-1_CRF01_AE_ and HIV-1_JR-FL_, respectively. Statistical significance was determined through an unpaired two-tailed Mann-Whitney *t*-test, and *P* values <0.05 were considered statistically significant. In both panels, a dotted line indicates the limit of detection, which was determined using five plasmas from uninfected individuals. The characteristics of the cohort of plasma donors are shown in Table 1.

We made a direct comparison of the susceptibility of infected cells to ADCC using representative infectious molecular clones (IMCs) from CRF01_AE (strain 703357) and subtype B (strain JR-FL). First, we evaluated the binding capacity of plasma from 10 PLWH infected by clade B viruses (Table 1). No significant differences between the two strains were observed (Fig. 1A). However, the ADCC responses to HIV-1_CRF01_AE_ were approximately twofold higher than those observed with the HIV-1_JR-FL_ strain (Fig. 1B). To evaluate whether the infecting HIV subtype could impact the functional differences observed, we also tested plasma from 10 PLWH infected by CRF01_AE viruses from the Thai RV304 cohort (Table 1). Interestingly, we observed a significant increase in both plasma binding and ADCC responses to HIV-1_CRF01_AE_ compared with HIV-1_JR-FL_ strain (Fig. S1).

**TABLE 1:** Characteristics of the cohorts of PLWH

Donor	HIV subtype	Sex	Age (years)	Days since infection	Days between inf. and ART	Viral load (copies/mL)	CD4 count (cells/mm^3^)
P5	B	M	33	936	192	50	1,149
P8	B	M	42	999	N/A^*a*^	28,666	260
P10	B	M	58	961	204	50	170
P13	B	M	28	1,194	N/A	809,600	200
P16	B	F	36	1,576	833	50	570
P25	B	M	33	1,173	N/A	35,922	421
P30	B	M	40	1,143	N/A	34,759	691
P43	B	M	34	856	N/A	29,234	410
P44	B	M	34	1,018	534	40	600
P46	B	M	56	1,005	986	40	780
RV304_29	CRF01_AE	M	21	N/A	N/A	63,588	227
RV304_31	CRF01_AE	M	47	N/A	N/A	40	267
RV304_43	CRF01_AE	M	28	N/A	N/A	40	1,067
RV304_50	CRF01_AE	M	28	N/A	N/A	7,452	283
RV304_51	CRF01_AE	M	47	N/A	N/A	40	565
RV304_53	CRF01_AE	M	23	N/A	N/A	40	923
RV304_62	CRF01_AE	M	24	N/A	N/A	6,801	470
RV304_65	CRF01_AE	M	25	N/A	N/A	40	1,131
RV304_66	CRF01_AE	M	28	N/A	N/A	57,167	226
RV304_80	CRF01_AE	M	31	N/A	N/A	194,000	208

^*a*^ N/A, not available.

**Fig 2:**
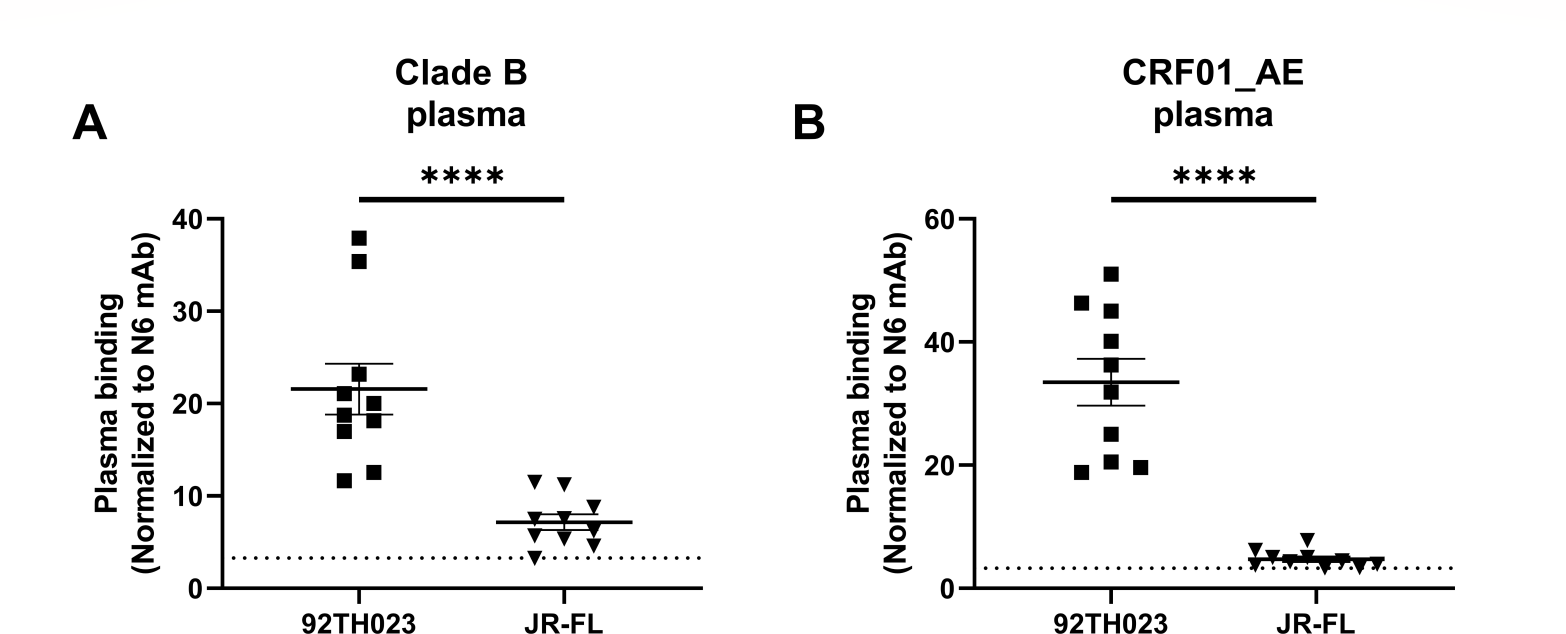
Plasma binding against 293T cells transfected with Env from CRF01_AE_92TH023_ or JRFL using plasma from PLWH infected with (**A**) clade B or (**B**) CRF01_AE strains. In both panels, a dotted line indicates the limit of detection, which was determined using five plasmas from uninfected individuals. Data shown are representative of two independent experiments. Asterisks indicate *P* < 0.0001.

To further explore the intrinsic Env conformational landscape of HIV-1_CRF01_AE_ and HIV-1_JR-FL_, we decided to evaluate the binding capacity of plasma from both cohorts (Table 1) in a CD4-negative cell line. Briefly, HEK293T cells were transfected with plasmids encoding the Env from CRF01_AE (strain 92TH023) or subtype B (strain JR-FL), and plasma binding was measured using flow cytometry. In concordance with our results obtained in primary CD4 T cells, we observed for both cohorts a significant increase in plasma binding against HIV-1_CRF01_AE_ compared to HIV-1_JR-FL_ (Fig. 2). Because activation of the ADCC response has been associated with a conformation of HIV-1 Env that enables binding of a specific class of Abs, these results suggest that HIV-1_CRF01_AE_ Env may have distinct conformational features that confer sensitivity to ADCC (6, 7).

### Modifications in HIV-1_CRF01_AE_ Env that enable site-specific fluorescent labeling do not affect viral infectivity

**Fig 3:**
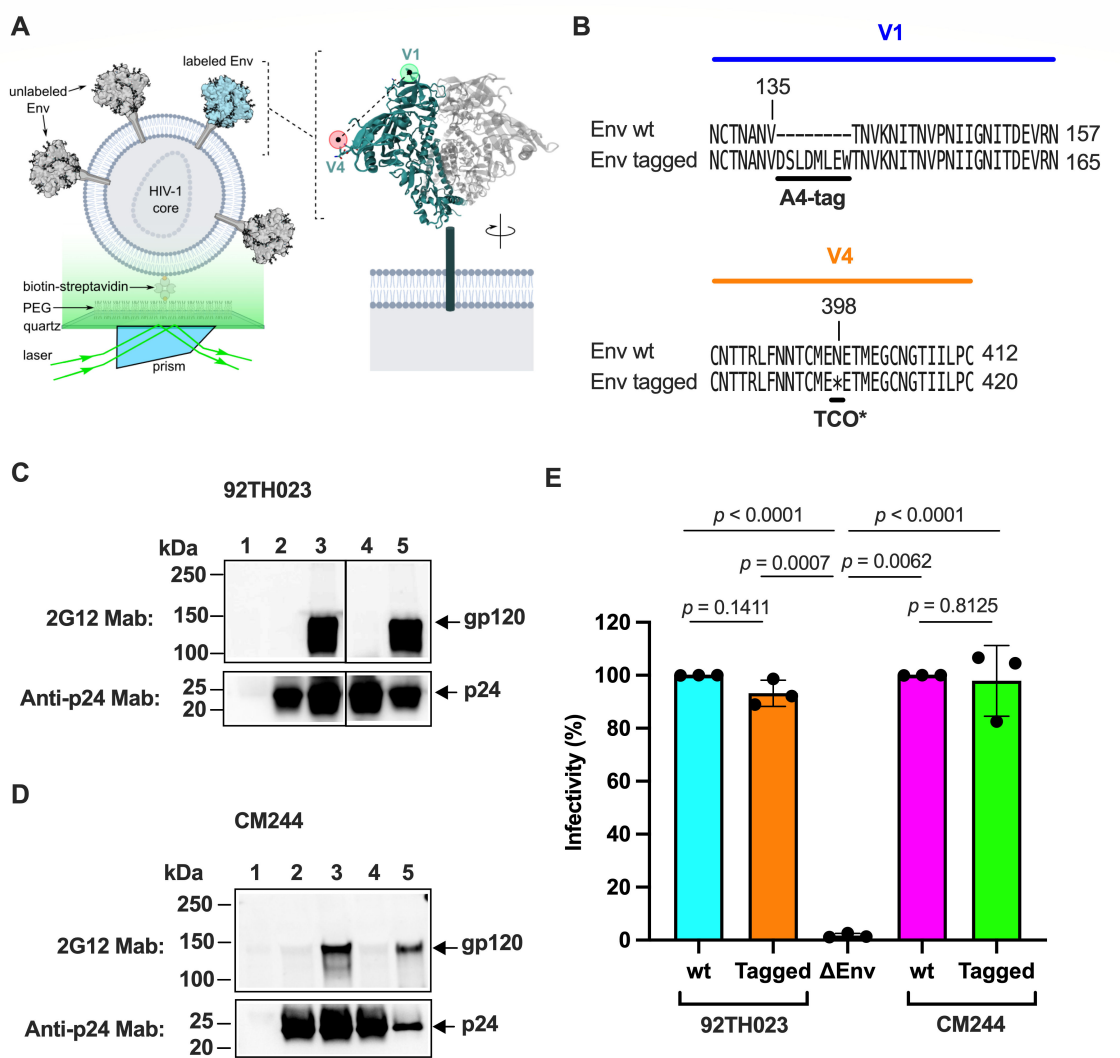
Engineering HIV-1_CRF01_AE_ Env for site-specific fluorescent labeling. (**A**) Schematic of the smFRET imaging assay. Pseudovirions with HIV-1_CRF01_AE_ Env (strain 92TH023) containing a single labeled gp120 domain were immobilized on quartz slides and imaged using total internal reflection fluorescence (TIRF) microscopy. (**B**) Sequence alignments indicating sites of A4 peptide insertion into the V1 loop and TCO* substitution in the V4 loop for fluorophore attachment. (**C**) Qualitative detection of the indicated proteins from purified pseudovirions with HIV-1_92TH023_ Env through immunoblots. Lane 1, mock-produced virus; lane 2, ΔEnv virions; lane 3, wild-type Env pseudotyped virions; lane 4, Env V1-A4/V4-TAG (tagged) pseudotyped virions produced in the absence of the TCO* amino acid; lane 5, tagged Env pseudovirions produced in the presence of the TCO* amino acid. (**D**) The same immunoblots but with HIV-1_CM244_ Env. (**E**) Infectivity of lentiviruses with wild-type HIV-1_92TH023_ or HIV-1_CM244_ Env (wt), with or without V1-A4/V4-TAG tags (tagged), or bald particles (ΔEnv) was evaluated in TZM-bI cells. Infectivity values are expressed as the percentage of the respective wild-type Env and normalized to the expression level of gp120 and p24. Each point indicates the arithmetic mean of three technical replicates. Bars represent the average of three independent experiments per condition. Error bars reflect the standard error. The statistical significance was evaluated through parametric *t*-tests. *P* values are indicated, and those <0.05 were considered statistically significant.

With the aim of visualizing the conformational dynamics of HIV-1_CRF01_AE_ Env, we adapted a previously validated smFRET imaging assay. We investigated CRF01_AE strains 92TH023 and CM244, which are tier-1 and tier-2 isolates, respectively. Insertion of the A4 peptide (DSLDMLEW) and incorporation of non-natural amino acids (nnAAs) into HIV-1 Env facilitate fluorophore attachment. These methods have been applied with minimal effect on functionality to subtype-B HIV-1 strains NL4-3 and JR-FL, as well as the subtype-A strain BG505 (8–10, 12, 30). As for previous applications, we attached site-specifically fluorophores in the V1 and V4 loops of a single gp120 domain within CRF01_AE Env on the surface of pseudovirions (Fig. 3A). To this end, we inserted the A4 peptide next to V135 in V1 (V1-A4), which enabled enzymatic attachment of the LD650 fluorophore. We also substituted an amber stop codon for amino acid N398 in V4 of gp120 (V4-N398^TAG^). Suppression of the amber stop codon incorporates the nnAA TCO*, which facilitated Cy3 fluorophore attachment through copper-free click chemistry (Fig. 3B) (34).

We next confirmed full-length translation of HIV-1_92TH023_ and HIV-1_CM244_ Env containing the V1-A4 and V4-N398^TAG^ mutations (tagged) and its incorporation into virions. We evaluated through immunoblots the abundance of both full-length gp120 and the HIV-1 core capsid protein p24 in purified viral preparations (Fig. 3C and D). As expected, tagged gp120 was not detected in virions produced in the absence of the nnAA TCO* and the corresponding aminoacyl tRNA synthetase and suppressor tRNA, which codes for the amber stop codon. This indicates that readthrough of the amber codon in the V4 loop did not occur, resulting in the lack of Env incorporation into viral particles (Fig. 3C and D, top immunoblots, lane 4). However, in the presence of TCO*, the synthetase, and the suppressor tRNA, tagged gp120 was detected in virions at a comparable level as wild-type Env (Fig. 3C and D, top immunoblots, lane 5). We next verified that V1-A4/V4-N398^TAG^ modifications in the Envs do not alter virus infectivity. Virus preparations bearing wild-type or tagged Env showed no statistically significant difference in their infectivity in TZM-bI cells (Fig. 3E), suggesting that both incorporation of the A4 peptide in V1 and the nnAA TCO* in V4 do not affect the function of Env. Altogether, these data demonstrate that tagged HIV-1_92TH023_ and HIV-1_CM244_ Envs are incorporated into pseudovirions and maintain native function during infection of cells.

### Native CRF01_AE HIV-1 Envs intrinsically sample “open” conformations

**Fig 4:**
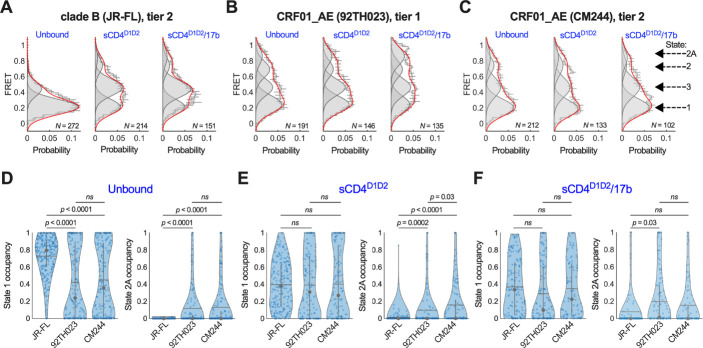
Conformational equilibrium of HIV-1 Env from clade B and CRF01_AE. (**A**) FRET histograms from unbound HIV-1_JR-FL_ Env trimers, or Env pre-incubated with sCD4^D1D2^, or sCD4^D1D2^ and 17b, as indicated. FRET histograms are presented as the mean ± standard error determined from three technical replicates, and the total number of smFRET traces used in the HMM analysis is shown (*N*). Overlaid on the histograms are four Gaussian distributions shown in gray and centered at 0.22 (State 1), 0.45 (State 3), 0.70 (State 2), and 0.85 (State 2A) FRET as determined through HMM analysis. The sum of the four Gaussians is shown in red. (**B**) The same data acquired for HIV-1_92TH023_ Env and (**C**) HIV-1_CM244_ Env. (**D**) Violin plots indicating the distribution in State 1 and State 2A occupancies seen for the population of individual Env molecules. The horizontal gray line indicates the mean occupancy; the gray circle and vertical whiskers indicate the median and quantiles, respectively. *P* values were determined through 1-way ANOVA and multiple comparison test. *P* > 0.05 was taken to be not significant (ns). (**E**) The same FRET state occupancy data Envs in the presence of (**E**) sCD4^D1D2^, or (**F**) sCD4^D1D2^ and 17b. Numeric occupancies of all four FRET states are shown in Table 2.

We next sought to evaluate the conformational dynamics in real-time of individual HIV-1_92TH023_ and HIV-1_CM244_ Env molecules on the surface of virions using smFRET imaging. To this end, we prepared virions bearing a single fluorescently labeled gp120 domain as described for Env from other HIV-1 strains (Fig. 3A) (8–10, 12). Labeled virions were immobilized on passivated quartz microscope slides and imaged using prism-based TIRF microscopy. We used the well-characterized clade-B HIV-1_JR-FL_ Env as a point of comparison. Consistent with previous reports, the application of hidden Markov modeling (HMM) for analysis of the smFRET trajectories enabled the identification of four FRET states, as indicated by the four Gaussian fits overlaid on the FRET histograms (Fig. 4A through C). HMM also allowed us to determine the fraction of time (occupancy) each molecule spent in each state (Table 2), which are presented as violin plots (Fig. 4D through F). The violin plot representation displays the heterogeneity of the population and allows for statistical comparisons across the data sets. For all three strains under consideration—HIV-1_JR-FL_, HIV-1_92TH023_, and HIV-1_CM244_—a low-FRET value (0.22 ± 0.1 FRET [mean ± standard deviation], State 1) was predominant, which is associated with a “closed” Env conformation (Fig. 4A through C). Quantification of the mean occupancies in State 1 across the populations of molecules indicated 72% ± 2%, 42% ± 2%, and 45% ± 2% for HIV-1_JR-FL_, HIV-1_92TH023_, and HIV-1_CM244_, respectively (Fig. 4D). We also observed State 3 (0.45 ± 0.1 FRET) for all strains, which is associated with an “open” Env conformation. We determined State 3 occupancies of 26% ± 2%, 27% ± 2%, and 28% ± 2% for HIV-1_JR-FL_, HIV-1_92TH023_, and HIV-1_CM244_, respectively (Table 2). Consistent with previous reports, we detected minimal occupancy for HIV-1_JR-FL_ Env in States 2 and 2A (0.70 ± 0.1 and 0.85 ± 0.1 FRET, respectively). In contrast, both unliganded CRF01_AE strains displayed modest but statistically significant occupancies in State 2 and State 2A. HIV-1_92TH023_ displayed 19% ± 1% occupancy in State 2 and 12% ± 1% in State 2A; HIV-1_CM244_ displayed 14% ± 1% and 13% ± 1% in State 2 and State 2A, respectively. These data demonstrate that Envs from both tier-1 and tier-2 strains of CRF01_AE have greater intrinsic access to “open” conformations than HIV-1_JR-FL_ Env.

**TABLE 2:** FRET-state occupancies for HIV-1 Env in the presence and absence of sCD4^D1D2^ and 17b

Subtype, strain	Experimental condition	FRET state occupancy (%)*^a^*			
		State 1	State 3	State 2	State 2A
Clade B, JR-FL	Unbound	73 ± 2	26 ± 2	1 ± 1	0 ± 0
	+ sCD4^D1D2^	39 ± 2	46 ± 2	13 ± 2	1 ± 1
	+ sCD4^D1D2^/17b	37 ± 3	42 ± 2	13 ± 2	8 ± 2
CRF01_AE, 92TH023	Unbound	42 ± 3	27 ± 2	19 ± 1	12 ± 1
	+ sCD4^D1D2^	39 ± 3	33 ± 2	18 ± 2	10 ± 2
	+ sCD4^D1D2^/17b	29 ± 3	34 ± 2	21 ± 2	22 ± 2
CRF01_AE, CM244	Unbound	45 ± 2	28 ± 2	14 ± 1	13 ± 1
	+ sCD4^D1D2^	40 ± 3	28 ± 2	16 ± 2	15 ± 2
	+ sCD4^D1D2^/17b	35 ± 3	34 ± 3	17 ± 2	14 ± 3

^*a*^ Data are presented as mean ± standard error determined from the total population of traces analyzed.

We next asked if sCD4 consisting of domains 1 and 2 (sCD4^D1D2^) or the anti-CoRBS mAb 17b further stabilize “open” conformations. For all three Envs, the addition of sCD4^D1D2^ destabilized State 1 and promoted transition to the higher FRET states, with the effect being the greater for HIV-1_JR-FL_. Although no statistically significant difference was seen in the State 1 occupancy across the three stains (Fig. 4E; Table 2). For HIV-1_JR-FL_ Env, we observed increased occupancy in States 2 and 3, as previously reported (12). sCD4^D1D2^ had only a modest effect on the conformations of both CRF01_AE Envs, with only a slight stabilization of State 3. The State 2A occupancy of HIV-1_92TH023_ and HIV-1_CM244_ Envs changed minimally in the presence of sCD4^D1D2^ but remained greater than seen for HIV-1_JR-FL_.

The addition of both sCD4^D1D2^ and 17b further promoted State 3 for HIV-1_JR-FL_ Env, as expected (Fig. 4F; Table 2). In contrast, the predominant effect of sCD4^D1D2^/17b on the tier-1 HIV-1_92TH023_ Env was to stabilize State 2A, increasing the occupancy to 22% ± 2%, as compared to 8% ± 2% for HIV-1_JR-FL_. In contrast, the tier-2 HIV-1_CM244_ showed no significant change in the State 2A occupancy with sCD4^D1D2^/17b, although it again remained greater than HIV-1_JR-FL_. Access to State 2A correlates with the greater inherent sensitivity to ADCC seen for HIV-1 from CRF01_AE.

## DISCUSSION

During HIV-1 infection, the humoral response against Env mainly produces antibodies that are non-neutralizing. Despite the lack of neutralization, nnAbs can still trigger ADCC to clear infected cells, provided that Env is exposed in an “open” conformation (35). Env glycoproteins from most HIV-1 strains naturally adopt State 1, which is associated with a closed conformation (12), and confers resistance to nnAbs (23, 36). In contrast, previous functional studies suggested that Env glycoproteins from CRF01_AE strains intrinsically adopt “open” conformations even in the absence of CD4, CD4 mimetics, or anti-CoRBS mAbs (6, 7, 13). Recent insights from structural data further support this idea (14). Here, we have shown that plasma obtained from PLWH triggers ADCC against CRF01_AE HIV-1-infected cells to a greater extent than the clade-B HIV-1_JR-FL_-infected cells. We therefore sought to directly test the conformational equilibrium of CRF01_AE HIV-1 Env using smFRET imaging. We have demonstrated through real-time analysis of HIV-1_92TH023_ and HIV-1_CM244_ Env conformational dynamics that these glycoproteins intrinsically sample “open” conformations in the absence of bound ligands. The tier-1 HIV-1_92TH023_ appeared to be slightly less stable in State 1 as compared to the neutralization-resistant tier-2 HIV-1_CM244_, consistent with greater exposure of antigenic sites in tier-1 isolates. However, this difference failed to reach statistical significance. Both CRF01_AE Envs intrinsically adopted State 2A, which was previously linked to exposure of both the CoRBS and cluster A epitopes that are targeted by Abs with potent ADCC activity (9). This provides a mechanistic rationale for the enhanced sensitivity to ADCC seen in the presence of plasma from PLWH. Only in the presence of both sCD4^D1D2^ and 17b was State 2A stabilized on HIV-1_92TH023_ Env, whereas the State 2A occupancy on HIV-1_CM244_ Env remained similar to unbound Env. Here again, this probably reflects the increased concealment of antigenic sites on the tier-2 isolate.

The data presented here provide a means of interpreting the inherent sensitivity of CRF01_AE HIV-1 to ADCC in terms of the conformation of Env. These data also provide a new understanding of the role of vaccine-induced Abs that mediated ADCC during the RV144 trial in Thailand, where HIV-1_CRF01_AE_ predominates (6). These data underscore the importance of considering Env conformational diversity across different HIV-1 clades when designing more effective HIV-1 interventions and vaccine strategies. This is of particular importance for the development of tailored strategies for enhancing ADCC against CRF01_AE HIV-1, which offers promising avenues for the elimination of cells infected with this prevalent strain in Southeast Asia.

## MATERIALS AND METHODS

### Plasma samples

The FRQS-AIDS and Infectious Diseases Network supports a representative cohort of newly HIV-infected subjects with clinical indication of primary infection (the Montreal Primary HIV Infection Cohort). Plasma samples from 10 deidentified PLWH donors were heat-inactivated and stored at -80°C (24, 26). The RV304/SEARCH 013 (NCT00796263) supports a cohort from Bangkok, Thailand who initiated antiretroviral therapy during the chronic phase of infection (CHI) (37). Plasma samples from 10 PLWH donors were heat-inactivated and stored at -80°C until use.

### Cell lines and primary cells

ExpiCHO-S cells (Gibco, Thermo Fisher Scientific, Waltham, MA, USA) were cultured in ExpiCHO Expression media (Gibco, Thermo Fisher Scientific, Waltham, MA, USA) at 37°C, 8% CO_2_ with orbital shaking according to the manufacturer’s instructions. HEK293T human embryonic kidney cells (obtained from ATCC) were grown as previously described (38). The cell line HEK293T-FIRB with enhanced furin expression was a kind gift from Dr. Theodore C. Pierson (Emerging Respiratory Virus section, Laboratory of Infectious Diseases, NIH, Bethesda, MD, USA), and was cultured at 37°C, 5% CO_2_ in complete DMEM made of DMEM (Gibco, Thermo Fisher Scientific, Waltham, MA, USA) supplemented with 10% (vol/vol) cosmic calf serum (Hyclone, Cytiva Life Sciences, Marlborough, MA, USA), 100 U/mL penicillin, 100 µg/mL streptomycin, and 1 mM glutamine (Gibco, Thermo Fisher Scientific, Waltham, MA, USA) (39). The use of HEK293T-FIRB cells minimizes the presence of uncleaved Env on the virions. The HeLa-derived TZM-bl cell line stably expressing high levels of CD4 and CCR5 receptors and bearing an integrated copy of the luciferase gene under the control of the HIV-1 long-terminal repeat (LTR) was obtained from the former NIH AIDS Reagent Program (BEI catalog HRP-8129) and cultured in the same conditions as HEK293T-FIRB cells (40).

Human peripheral blood mononuclear cells (PBMCs) from three HIV-negative individuals (three males, age range: 40–66 years) obtained by leukapheresis and Ficoll density gradient isolation were cryopreserved in liquid nitrogen until further use. Primary CD4+ T cells were purified from resting PBMCs by negative selection using immunomagnetic beads per the manufacturer’s instructions (StemCell Technologies, Vancouver, BC) and were activated with phytohemagglutinin-L (PHA-L, 10 µg/mL) for 48 h and then maintained in RPMI 1640 (Thermo Fisher Scientific, Waltham, MA, USA) complete medium supplemented with 20% FBS, 100 U/mL penicillin/streptomycin and with recombinant IL-2 (rIL-2, 100 U/mL). All cells were maintained at 37°C under 5% CO_2_.

### Plasmids and proviral constructs

The plasmid encoding the soluble CD4 domains 1 and 2 (sCD4^D1D2^) fused to an anti-6×-His tag, as well as the molecular clones of the heavy and light chains of the anti-HIV-1 Env monoclonal antibodies 17b and 2G12, were kindly provided by Dr. Peter Kwong (NIAID, NIH). Plasmids for expression of NESPylRS^AF^/hU6tRNA^Pyl^ and eRF1-E55D for the *amber* codon suppression system were previously described (34). The pNL4-3 Δ*RT* Δ*env* plasmid has been previously described (12). pNL4-3.Luc.*R-E*- provirus was obtained from the former NIH AIDS Reagent Program (BEI catalog HRP-3418). The stop codon in the *tat* gene of this plasmid was substituted with an *ochre* stop codon as described (41). Plasmids for the expression of full-length HIV-1_JR-FL_ Env wild type, which was engineered to have an amber (TAG) stop codon at position N135 in the V1 loop of gp120 and the A1 peptide (GDSLDMLEWSLM) in the V4 loop of gp120 (V1-N135^TAG^/V4-A1) have been previously described (30). The HIV-1_CRF01_AE_ Env expressors from strains 92TH023 and CM244 have been described (13). These plasmids were engineered to insert the A4 peptide (DSLDMLEW) after residue V135 in the V1 loop of gp120, and substitute an amber codon at position N398 in the V4 loop of gp120 (A4-V1/V4-N398^TAG^, Fig. 3B). All the indicated residues in HIV-1_JR-FL_ and HIV-1_CRF01_AE_ Env are numbered according to the HIV-1_HXBc2_ Env sequence.

The IMC of HIV-1_JR-FL_ was kindly provided by Dr. Dennis Burton (The Scripps Research Institute). The CRF01_AE IMC was previously reported (6). The sequence of HIV-1_CRF01_AE_ transmitted-founder (T/F) clone 703357 was derived by using a single-genome amplification strategy. The entire DNA sequence, including both LTRs, was cloned into pUC57 to generate a full-length IMC (GenBank accession numbers: JX448154 and JX448164). The vesicular stomatitis virus G (VSV-G)-encoding plasmid was previously described (24).

### Recombinant sCD4^D1D2^ and antibodies

Expression of soluble CD4 domains D1-D2 (sCD4^D1D2^) fused to an anti-6×-His tag was performed by transfection of ExpiCHO-S cells with plasmid using the ExpiFectamine CHO Transfection Kit (Gibco, Thermo Fisher Scientific, Waltham, MA, USA) according to the manufacturer’s instructions. Purification and preparation of this protein were performed with a previously described strategy (42). Briefly, supernatant containing soluble sCD4^D1D2^ was harvested 9 days post-transfection and adjusted to 1 mM NiSO_4_, 20 mM imidazole, and pH 8.0 before binding to the Ni-NTA resin (Invitrogen, Waltham, MA, USA). The resin was washed, and sCD4^D1D2^ was eluted from the column with 300 mM imidazole, 500 mM NaCl, 20 mM Tris-HCl pH 8.0, and 10% (vol/vol) glycerol. Elution fractions containing sCD4^D1D2^ were pooled and concentrated by centrifugal concentrators (Sartorius AG, Göttingen, Germany). Final purification was performed through size exclusion chromatography on a Superdex 200 Increase 10/300 GL column (GE Healthcare, Chicago, IL, USA) followed by concentration as described above.

Expression and preparation of monoclonal antibodies 2G12 and 17b have been described before (42, 43). Briefly, ExpiCHO-S cells were co-transfected with plasmids encoding heavy and light chains using the ExpiFectamine CHO Transfection Kit (Gibco, Thermo Fisher Scientific, Waltham, MA, USA) according to the manufacturer’s instructions. Both antibodies were purified from the cell culture supernatant 12 days post-transfection using protein G affinity resin (Thermo Fisher Scientific, Waltham, MA, USA), subjected to buffer exchange with phosphate-buffered saline (PBS) pH 7.4 (Fisher Bioreagents, Thermo Fisher Scientific, Waltham, MA, USA) and concentrated as described above. Mouse monoclonal antibody targeting HIV-1 p24 capsid protein (anti-p24, catalog no. GTX41618) was purchased from Genetex (Irvine, CA, USA). Anti-6×-His-tag polyclonal antibody (catalog no. PA1-983B), horseradish peroxidase (HRP) conjugated anti-human IgG Fc (catalog no. A18823), and anti-mouse IgG Fc (catalog no. 31455) were purchased from Invitrogen (Waltham, MA, USA). Goat anti-rabbit IgG antibody conjugated to HRP (catalog no. ab205718) was purchased from Abcam (Cambridge, UK).

### Virus production and fluorescent labeling

Non-replicative HIV-1_CRF01_AE_ Env pseudoviruses for infectivity assays were produced by co-transfecting HEK293T-FIRB cells with either a 1:0.005 or 1:1 mass ratio of plasmid pNL4-3.Luc.R-E- *tat*_*ochre* to wild-type or V1-A4/V4-N398^TAG^ tagged version of HIV-1_CRF01_AE_ Env expressors, respectively. Plasmids encoding NESPylRS^AF^/hU6tRNA^Pyl^ and eRF1-E55D were also included along with 0.5 mM TCO* (SiChem GmbH, Bremen, Germany) as previously described (30, 41, 44, 45). Virus was collected 48 h post-transfection and pelleted over a 10% sucrose cushion at 25,000 RPM for 2 h at 4°C using an SW32Ti rotor (Beckman Coulter Life Sciences, Brea, CA, USA). Pellets were resuspended in DMEM (Gibco, Thermo Fisher Scientific, Waltham, MA, USA), aliquoted, and stored at −80°C until use.

For smFRET imaging, non-replicative HIV-1_JR-FL_ and HIV-1_CRF01_AE_ Env pseudovirions with a single gp120 domain bearing the above-mentioned modifications in the V1 and V4 loops were also produced in the presence of TCO* as previously described (30). Briefly, HEK-293T FIRB cells were co-transfected with plasmids NESPylRS^AF^/hU6tRNA^Pyl^ and eRF1-E55D, in addition to pNL4-3 ΔRT ΔEnv, and a 20:1 mass ratio of HIV-1_JR-FL_ or HIV-1_CRF01_AE_ Env wild-type expressor to the corresponding tagged version. The virus was collected 48 h post-transfection and pelleted as above. Virus pellets were then resuspended in labeling buffer (50 mM HEPES pH 7.0, 10 mM CaCl_2_, 10 mM MgCl_2_), and incubated overnight at room temperature with 5 µM LD650-coenzyme A (Lumidyne Technologies, New York, NY, USA), and 5 µM acyl carrier protein synthase (AcpS), which labels the A1 (or A4) peptide. The virus was then incubated with 0.5 µM Cy3-tetrazine (Jena Biosciences, Jena, Germany) for 30 min at room temperature, followed by incubation with 60 µM DSPE-PEG2000-biotin (Avanti Polar Lipids, Alabaster, AL, USA) for an additional 30 min at room temperature. Finally, the labeled virus was purified through ultracentrifugation for 1 h at 35,000 RPM using a rotor SW40Ti (Beckman Coulter Life Sciences, Brea, CA, USA), at 4°C in a 6%–30% OptiPrep (Sigma-Aldrich, MilliporeSigma, Burlington, MA, USA) density gradient. Labeled pseudovirions were collected, analyzed by anti-p24 Western blot, aliquoted, and stored at −80°C until their use in imaging experiments.

### Immunoblots

HIV-1 gp120 and p24 proteins, or sCD4^D1D2^, were detected through immunoblot assays as follows. Samples were mixed with 4× Laemmli sample buffer (Bio-Rad, Hercules, CA, USA) supplemented with 2-mercaptoethanol (Fisher Chemical, Hampton, NH, USA) and heated for 5 min at 98°C. Proteins were then resolved by denaturing PAGE using 4%–20% acrylamide gels (Bio-Rad, Hercules, CA, USA). Proteins were then transferred to nitrocellulose membranes (Bio-Rad, Hercules, CA, USA) according to the manufacturer’s instructions. After blocking for 1 h at room temperature with 5% (wt/vol) skim milk in PBS-T buffer (PBS and 0.1% [vol/vol] Tween-20, Fisher Scientific, Hampton, NH, USA), membranes were incubated overnight at 4°C with the indicated primary antibodies diluted in blocking buffer. Detection of gp120 was achieved by using a 3 µg/mL dilution of 2G12, while detection of p24 and sCD4^D1D2^ was performed with 2 µg/mL dilutions of anti-p24 mAb (GeneTex, Irvine, CA, USA) or rabbit anti-6×-His-tag polyclonal antibody (Invitrogen, Waltham, MA, USA), respectively. Membranes were washed three times with PBS-T and incubated for 1 h at room temperature with a 1/10,000 dilution (vol/vol) in 0.5% (wt/vol) skim milk/PBS-T of HRP-conjugated anti-human IgG Fc or anti-mouse IgG Fc (Invitrogen, Waltham, MA, USA) antibodies for membranes incubated with 2G12 or anti-p24 mAbs, respectively, or a 1/50,000 dilution of HRP-conjugated anti-rabbit IgG antibody (Abcam, Cambridge, UK) was used for membranes incubated with anti-6×-His antibody. After three washes with PBS-T, membranes were developed using SuperSignal West Pico PLUS Chemiluminescent Substrate (Thermo Scientific, Waltham, MA, USA) according to the manufacturer’s instructions. Due to the use of HEK293T-FIRB cells for virus production, we detected no uncleaved Env (gp160) for either of the CRF01_AE strains evaluated.

### Infectivity assays

TZM-bl cells (2.5 × 10^4^/well) were seeded 24 h before the assay in 24-well plates. Cells were then washed once with DMEM (Gibco, Thermo Fisher Scientific, Waltham, MA, USA) and inoculated with pseudo-typed lentiviruses bearing wild-type or tagged HIV-1_CRF01_AE_ Env. After 2 h of virus adsorption at 37°C, viral inoculums were removed, and cells were washed with DMEM, followed by the addition of fresh complete phenol red-free DMEM (Gibco, Thermo Fisher Scientific, Waltham, MA, USA). Cell supernatants were removed 48 h post-infection. The cells were lysed with Glo Lysis Buffer (Promega, Madison, WI, USA) according to the manufacturer’s instructions. Luciferase activity in cell lysates was detected by mixing equal volumes of lysate and Steady-Glo Luciferase Assay System reagent (Promega, Madison, WI, USA) and measured on a Synergy H1 microplate reader (Biotek, Winooski, VT, USA). The luminescence signal from mock-infected cell lysates was subtracted from the signal obtained from infected cells and normalized by the abundance of both envelope gp120 and p24 proteins in viral inoculums, which were determined through densitometric analysis of protein bands observed in immunoblots using ImageJ software v1.52q (NIH, Bethesda, MD, USA). Infectivity was expressed as the percentage of that seen in cells inoculated with wild-type HIV-1_CRF01_AE_ Env pseudovirions.

### smFRET imaging

Labeled HIV-1_JR-FL_ or HIV-1_CRF01_AE_ Env pseudovirions were immobilized on streptavidin-coated quartz slides and imaged on a custom-built wide-field prism-based TIRF microscope (41, 46). Where indicated, pseudovirions were incubated with 50 µM sCD4^D1D2^ and 50 µg/mL 17b mAb for 1 h at room temperature prior to surface immobilization. Imaging was performed in PBS pH ~ 7.4, containing 1 mM trolox (Sigma-Aldrich, St. Louis, MO, USA), 1 mM cyclooctatetraene (Sigma-Aldrich, St. Louis, MO, USA), 1 mM 4-nitrobenzyl alcohol (Sigma-Aldrich, St. Louis, MO, USA), 2 mM protocatechuic acid (Sigma-Aldrich, St. Louis, MO, USA), and 8 nM protocatechuate 3,4-dioxygenase (Sigma-Aldrich, St. Louis, MO, USA) to stabilize fluorescence and remove molecular oxygen. When indicated, concentrations of sCD4^D1D2^ and mAb 17b were maintained during imaging. smFRET data were collected using Micromanager v2.0 at 25 frames/s, processed, and analyzed using SPARTAN software in Matlab (Mathworks, Natick, MA, USA) (47). smFRET traces were identified according to criteria previously described (9); traces meeting those criteria were verified manually. FRET histograms were generated by compiling traces from each of three technical replicates, and the mean probability per histogram bin ± standard error was calculated. Traces were idealized to a five-state HMM (four nonzero-FRET states and a zero-FRET state) using the maximum point likelihood algorithm (48). The idealizations were used to construct Gaussian distributions of each FRET state, which were overlaid on the FRET histograms to visualize the results of the HMM analysis. The HMM analysis was also used to determine the occupancies (fraction of time until photobleaching) in each FRET state for each individual Env molecule. The distributions in occupancies were used to construct violin plots in Matlab, as well as the calculation of mean occupancies and standard errors. Whereas the FRET histograms reflect an estimate of the thermodynamics of the Env conformational equilibrium, the violin plots display the heterogeneity of the population and permit statistical comparisons (*P* values).

### Viral production and infection of primary CD4+ T cells

VSV-G-pseudotyped HIV-1 viruses were produced by co-transfection of 293T cells with the HIV-1_JRFL_ or HIV-1_CRF01_AE_ proviral construct and a VSV-G-encoding vector at a ratio of 3:2 using the polyethylenimine method. Two days post-transfection, cell supernatants were harvested, clarified by low-speed centrifugation (300 × *g* for 5 min), and concentrated by ultracentrifugation at 4°C (100,605 × *g* for 1 h) over a 20% sucrose cushion. Pellets were resuspended in fresh RPMI 1640 complete medium, aliquoted, and stored at −80°C until use.

Primary CD4+ T cells from HIV-1-negative individuals were isolated from PBMCs, activated for 2 days with PHA-L, and then maintained in RPMI 1640 complete medium supplemented with rIL-2. Five to seven days after activation, the cells were spinoculated with the virus at 800 × *g* for 1 h in 96-well plates at 25°C. All viral productions were titrated on primary CD4+ T cells to achieve similar levels of infection (around 15% of infected cells).

### Flow cytometry analysis of cell-surface staining

Forty-eight hours after infection, HIV-1-infected primary CD4+ T cells were collected, washed with PBS, and transferred to 96-well V-bottom plates. The cells were then incubated for 45 min at 37°C with plasma (1:1,000 dilution). Cells were then washed twice with PBS and stained with anti-human IgG Alexa Fluor 647-conjugated secondary antibody (2 µg/mL), FITC-conjugated mouse anti-human CD4 (Clone OKT4) antibody (1:500 dilution) and AquaVivid viability dye (1:1,000 dilution) (Thermo Fisher Scientific, Cat# L43957) for 20 min at room temperature. Cells were then washed twice with PBS and fixed in a 2% PBS-formaldehyde solution. The cells were then permeabilized using the Cytofix/Cytoperm Fixation/Permeabilization Kit (BD Biosciences, Mississauga, ON, Canada) and stained intracellularly using PE-conjugated mouse anti-p24 mAb (clone KC57; Beckman Coulter, Brea, CA, USA; 1:100 dilution). Samples were acquired on a Fortessa cytometer (BD Biosciences), and data analysis was performed using FlowJo v10.5.3 (Tree Star, Ashland, OR, USA). The percentage of productively infected cells (p24^+^, CD4 low) was determined by gating on the living cell population according to viability dye staining (AquaVivid; Thermo Fisher Scientific). Dotted lines represent the limit of detection calculated using five plasmas from uninfected individuals.

Cell surface staining of Env-expressing 293T cells was performed as previously described (7). Briefly, 2  ×  10^6^ cells were transfected with 7 µg of Env expressor and 1 µg of a green fluorescent protein (GFP) expressor (pIRES-GFP) with the calcium-phosphate method. At 48 h post-transfection, 293T cells were stained with anti-Env antibodies (5 µg/mL) or plasma at a 1:1,000 dilution. To normalize Env expression, we used the anti-CD4BS bNabs N6, known to potently neutralize 98% of circulating isolates, including CRF01_AE and clade B strains (49). As such, plasma binding (median fluorescent intensity [MFI]) was normalized to the MFI of N6 bNabs obtained for each respective Envs. Dotted lines represent the limit of detection calculated using five plasmas from uninfected individuals.

### ADCC assay

ADCC activity was measured using a FACS-based infected cell elimination assay 48 h after infection. The HIV-1-infected primary CD4+ T cells were stained with AquaVivid viability dye and cell proliferation dye eFluor670 (Thermo Fisher Scientific) and used as target cells. Resting autologous PBMCs were stained with cell proliferation dye eFluor450 (Thermo Fisher Scientific) and used as effector cells. The HIV-1-infected primary CD4+ T cells were co-cultured with autologous PBMCs (effector:target ratio of 10:1) in 96-well V-bottom plates in the presence of plasma from PLWH (dilution 1:1,000) for 5 h at 37°C. After the 5 h incubation, cells were then washed once with PBS and stained with FITC-conjugated mouse anti-human CD4 (Clone OKT4) antibody for 10 min at room temperature. Cells were then washed twice with PBS and fixed in a 2% PBS-formaldehyde solution. The cells were then permeabilized and stained intracellularly for p24 as described above. Samples were acquired on a Fortessa cytometer (BD Biosciences), and data analysis was performed using FlowJo v10.5.3 (Tree Star, Ashland, OR, USA). The percentage of infected cells (p24^+^, CD4 low) was determined by gating on the living cell population according to viability dye staining (AquaVivid; Thermo Fisher Scientific). The percentage of ADCC was calculated with the following formula: (% of p24^+^CD4 low cells in Targets plus Effectors) − (% of p24^+^CD4 low cells in Targets plus Effectors plus plasma)/(% of p24^+^CD4 low cells in Targets) × 100. Dotted lines represent the limit of detection calculated using five plasmas from uninfected individuals.

### Statistical analysis

Statistics for infectivity assays were determined using GraphPad Prism version 10.2.3 (GraphPad, San Diego, CA, USA). Every data set was tested for statistical normality, and this information was used to apply the appropriate (parametric or nonparametric) statistical test. Statistical significance measures (*P* values) of FRET state occupancies were determined by one-way ANOVA followed by multiple comparison testing in Matlab. In all cases, *P* values <0.05 were considered statistically significant.

## Data Availability

All data generated or analyzed during this study are included in the article.

## SUPPLEMENTAL MATERIAL

Figure S1 jvi.01667-25-s0001.pdf Plasma binding and ADCC responses.

ASM does not own the copyrights to Supplemental Material that may be linked to, or accessed through, an article. The authors have granted ASM a non-exclusive, world-wide license to publish the Supplemental Material files. Please contact the corresponding author directly for reuse.

## References

[1] Haynes BF, Gilbert PB, McElrath MJ, Zolla-Pazner S, Tomaras GD, Alam SM, Evans DT, Montefiori DC, Karnasuta C, Sutthent R, et al.. 2012. Immune-correlates analysis of an HIV-1 vaccine efficacy trial. N Engl J Med 366:1275–1286. doi:10.1056/NEJMoa1113425 22475592 PMC3371689

[2] Tomaras GD, Ferrari G, Shen X, Alam SM, Liao H-X, Pollara J, Bonsignori M, Moody MA, Fong Y, Chen X, Poling B, Nicholson CO, Zhang R, Lu X, Parks R, Kaewkungwal J, Nitayaphan S, Pitisuttithum P, Rerks-Ngarm S, Gilbert PB, Kim JH, Michael NL, Montefiori DC, Haynes BF. 2013. Vaccine-induced plasma IgA specific for the C1 region of the HIV-1 envelope blocks binding and effector function of IgG. Proc Natl Acad Sci USA 110:9019–9024. doi:10.1073/pnas.1301456110 23661056 PMC3670311

[3] Dai B, Peng X, Sun J, Zhu X, Liu X, Xiong Y, Wan Z, Xiang D, Hui J, Ying C, Liu H, Zhu B. 2024. Distinct clusters of HIV-1 CRF01_AE in Zhejiang, China: high-risk transmission cluster 4 requires heightened surveillance. Infect Drug Resist 17:4333–4342. doi:10.2147/IDR.S480192 39411499 PMC11476370

[4] Khairunisa SQ, Indriati DW, Megasari NLA, Ueda S, Kotaki T, Fahmi M, Ito M, Rachman BE, Hidayati AN, Kameoka M. 2024. Spatial-temporal transmission dynamics of HIV-1 CRF01_AE in Indonesia. Sci Rep 14:9917. doi:10.1038/s41598-024-59820-y 38730038 PMC11087524

[5] Hemelaar J, Elangovan R, Yun J, Dickson-Tetteh L, Kirtley S, Gouws-Williams E, Ghys PD, Abimiku AG, Agwale S, Archibald C, et al.. 2020. Global and regional epidemiology of HIV-1 recombinants in 1990-2015: a systematic review and global survey. Lancet HIV 7:e772–e781. doi:10.1016/S2352-3018(20)30252-6 33128904

[6] Prévost J, Zoubchenok D, Richard J, Veillette M, Pacheco B, Coutu M, Brassard N, Parsons MS, Ruxrungtham K, Bunupuradah T, Tovanabutra S, Hwang K-K, Moody MA, Haynes BF, Bonsignori M, Sodroski J, Kaufmann DE, Shaw GM, Chenine AL, Finzi A. 2017. Influence of the envelope gp120 phe 43 cavity on HIV-1 sensitivity to antibody-dependent cell-mediated cytotoxicity responses. J Virol 91:e02452-16. doi:10.1128/JVI.02452-16 28100618 PMC5355605

[7] Prévost J, Tolbert WD, Medjahed H, Sherburn RT, Madani N, Zoubchenok D, Gendron-Lepage G, Gaffney AE, Grenier MC, Kirk S, Vergara N, Han C, Mann BT, Chénine AL, Ahmed A, Chaiken I, Kirchhoff F, Hahn BH, Haim H, Abrams CF, Smith AB III, Sodroski J, Pazgier M, Finzi A. 2020. The HIV-1 env gp120 inner domain shapes the Phe43 cavity and the CD4 binding site. mBio 11:e00280-20. doi:10.1128/mBio.00280-20 32457241 PMC7251204

[8] Lu M, Ma X, Castillo-Menendez LR, Gorman J, Alsahafi N, Ermel U, Terry DS, Chambers M, Peng D, Zhang B, et al.. 2019. Associating HIV-1 envelope glycoprotein structures with states on the virus observed by smFRET. Nature 568:415–419. doi:10.1038/s41586-019-1101-y 30971821 PMC6655592

[9] Alsahafi N, Bakouche N, Kazemi M, Richard J, Ding S, Bhattacharyya S, Das D, Anand SP, Prévost J, Tolbert WD, Lu H, Medjahed H, Gendron-Lepage G, Ortega Delgado GG, Kirk S, Melillo B, Mothes W, Sodroski J, Smith III AI, Kaufmann DE, Wu X, Pazgier M, Rouiller I, Finzi A, Munro JB. 2019. An Asymmetric Opening of HIV-1 Envelope Mediates Antibody-Dependent Cellular Cytotoxicity. Cell Host Microbe 25:578–587. doi:10.1016/j.chom.2019.03.002 30974085 PMC6592637

[10] Ma X, Lu M, Gorman J, Terry DS, Hong X, Zhou Z, Zhao H, Altman RB, Arthos J, Blanchard SC, Kwong PD, Munro JB, Mothes W. 2018. HIV-1 env trimer opens through an asymmetric intermediate in which individual protomers adopt distinct conformations. eLife 7:e34271. doi:10.7554/eLife.34271 29561264 PMC5896952

[11] Herschhorn A, Ma X, Gu C, Ventura JD, Castillo-Menendez L, Melillo B, Terry DS, Smith AB III, Blanchard SC, Munro JB, Mothes W, Finzi A, Sodroski J. 2016. Release of gp120 restraints leads to an entry-competent intermediate state of the HIV-1 envelope glycoproteins. mBio 7:e01598-16. doi:10.1128/mBio.01598-16 27795397 PMC5080382

[12] Munro JB, Gorman J, Ma X, Zhou Z, Arthos J, Burton DR, Koff WC, Courter JR, Smith AB III, Kwong PD, Blanchard SC, Mothes W. 2014. Conformational dynamics of single HIV-1 envelope trimers on the surface of native virions. Science 346:759–763. doi:10.1126/science.1254426 25298114 PMC4304640

[13] Zoubchenok D, Veillette M, Prévost J, Sanders-Buell E, Wagh K, Korber B, Chenine AL, Finzi A. 2017. Histidine 375 modulates CD4 binding in HIV-1 CRF01_AE envelope glycoproteins. J Virol 91:e02151-16. doi:10.1128/JVI.02151-16 27928014 PMC5286895

[14] Prévost J, Chen Y, Zhou F, Tolbert WD, Gasser R, Medjahed H, Nayrac M, Nguyen DN, Gottumukkala S, Hessell AJ, Rao VB, Pozharski E, Huang RK, Matthies D, Finzi A, Pazgier M. 2023. Structure-function analyses reveal key molecular determinants of HIV-1 CRF01_AE resistance to the entry inhibitor temsavir. Nat Commun 14:6710. doi:10.1038/s41467-023-42500-2 37872202 PMC10593844

[15] Checkley MA, Luttge BG, Freed EO. 2011. HIV-1 envelope glycoprotein biosynthesis, trafficking, and incorporation. J Mol Biol 410:582–608. doi:10.1016/j.jmb.2011.04.042 21762802 PMC3139147

[16] Willey RL, Bonifacino JS, Potts BJ, Martin MA, Klausner RD. 1988. Biosynthesis, cleavage, and degradation of the human immunodeficiency virus 1 envelope glycoprotein gp160. Proc Natl Acad Sci USA 85:9580–9584. doi:10.1073/pnas.85.24.9580 2849111 PMC282803

[17] Earl PL, Doms RW, Moss B. 1990. Oligomeric structure of the human immunodeficiency virus type 1 envelope glycoprotein. Proc Natl Acad Sci USA 87:648–652. doi:10.1073/pnas.87.2.648 2300552 PMC53322

[18] Freed EO, Myers DJ, Risser R. 1989. Mutational analysis of the cleavage sequence of the human immunodeficiency virus type 1 envelope glycoprotein precursor gp160. J Virol 63:4670–4675. doi:10.1128/JVI.63.11.4670-4675.1989 2677400 PMC251101

[19] McCune JM, Rabin LB, Feinberg MB, Lieberman M, Kosek JC, Reyes GR, Weissman IL. 1988. Endoproteolytic cleavage of gp160 is required for the activation of human immunodeficiency virus. Cell 53:55–67. doi:10.1016/0092-8674(88)90487-4 2450679

[20] Wyatt R, Sodroski J. 1998. The HIV-1 envelope glycoproteins: fusogens, antigens, and immunogens. Science 280:1884–1888. doi:10.1126/science.280.5371.1884 9632381

[21] Allan JS, Coligan JE, Barin F, McLane MF, Sodroski JG, Rosen CA, Haseltine WA, Lee TH, Essex M. 1985. Major glycoprotein antigens that induce antibodies in AIDS patients are encoded by HTLV-III. Science 228:1091–1094. doi:10.1126/science.2986290 2986290

[22] Robey WG, Safai B, Oroszlan S, Arthur LO, Gonda MA, Gallo RC, Fischinger PJ. 1985. Characterization of envelope and core structural gene products of HTLV-III with sera from AIDS patients. Science 228:593–595. doi:10.1126/science.2984774 2984774

[23] Veillette M, Coutu M, Richard J, Batraville L-A, Dagher O, Bernard N, Tremblay C, Kaufmann DE, Roger M, Finzi A. 2015. The HIV-1 gp120 CD4-bound conformation is preferentially targeted by antibody-dependent cellular cytotoxicity-mediating antibodies in sera from HIV-1-infected individuals. J Virol 89:545–551. doi:10.1128/JVI.02868-14 25339767 PMC4301108

[24] Veillette M, Désormeaux A, Medjahed H, Gharsallah N-E, Coutu M, Baalwa J, Guan Y, Lewis G, Ferrari G, Hahn BH, Haynes BF, Robinson JE, Kaufmann DE, Bonsignori M, Sodroski J, Finzi A. 2014. Interaction with cellular CD4 exposes HIV-1 envelope epitopes targeted by antibody-dependent cell-mediated cytotoxicity. J Virol 88:2633–2644. doi:10.1128/JVI.03230-13 24352444 PMC3958102

[25] Richard Jonathan, Pacheco B, Gohain N, Veillette M, Ding S, Alsahafi N, Tolbert WD, Prévost J, Chapleau J-P, Coutu M, Jia M, Brassard N, Park J, Courter JR, Melillo B, Martin L, Tremblay C, Hahn BH, Kaufmann DE, Wu X, Smith AB III, Sodroski J, Pazgier M, Finzi A. 2016. Co-receptor binding site antibodies enable CD4-mimetics to expose conserved anti-cluster A ADCC epitopes on HIV-1 envelope glycoproteins. EBioMedicine 12:208–218. doi:10.1016/j.ebiom.2016.09.004 27633463 PMC5078604

[26] Richard J, Veillette M, Brassard N, Iyer SS, Roger M, Martin L, Pazgier M, Schön A, Freire E, Routy J-P, Smith AB III, Park J, Jones DM, Courter JR, Melillo BN, Kaufmann DE, Hahn BH, Permar SR, Haynes BF, Madani N, Sodroski JG, Finzi A. 2015. CD4 mimetics sensitize HIV-1-infected cells to ADCC. Proc Natl Acad Sci USA 112:E2687. doi:10.1073/pnas.1506755112 25941367 PMC4443331

[27] Prévost J, Richard J, Medjahed H, Alexander A, Jones J, Kappes JC, Ochsenbauer C, Finzi A. 2018. Incomplete downregulation of cd4 expression affects HIV-1 env conformation and antibody-dependent cellular cytotoxicity responses. J Virol 92:e00484-18. doi:10.1128/JVI.00484-18 29669829 PMC6002730

[28] Prévost J, Richard J, Ding S, Pacheco B, Charlebois R, Hahn BH, Kaufmann DE, Finzi A. 2018. Envelope glycoproteins sampling states 2/3 are susceptible to ADCC by sera from HIV-1-infected individuals. Virology (Auckl) 515:38–45. doi:10.1016/j.virol.2017.12.002 PMC5843759 29248757

[29] Rajashekar JK, Richard J, Beloor J, Prévost J, Anand SP, Beaudoin-Bussières G, Shan L, Herndler-Brandstetter D, Gendron-Lepage G, Medjahed H, et al.. 2021. Modulating HIV-1 envelope glycoprotein conformation to decrease the HIV-1 reservoir. Cell Host Microbe 29:904–916. doi:10.1016/j.chom.2021.04.014 34019804 PMC8214472

[30] Marchitto L, Richard J, Prévost J, Tauzin A, Yang D, Chiu T-J, Chen H-C, Díaz-Salinas MA, Nayrac M, Benlarbi M, Beaudoin-Bussières G, Anand SP, Dionne K, Bélanger É, Chatterjee D, Medjahed H, Bourassa C, Tolbert WD, Hahn BH, Munro JB, Pazgier M, Smith AB 3rd, Finzi A. 2024. The combination of three CD4-induced antibodies targeting highly conserved Env regions with a small CD4-mimetic achieves potent ADCC activity. J Virol 98:e0101624. doi:10.1128/jvi.01016-24 39248460 PMC11495009

[31] Anand SP, Prévost J, Baril S, Richard J, Medjahed H, Chapleau J-P, Tolbert WD, Kirk S, Smith AB III, Wines BD, Kent SJ, Hogarth PM, Parsons MS, Pazgier M, Finzi A. 2019. Two families of env antibodies efficiently engage fc-gamma receptors and eliminate HIV-1-infected cells. J Virol 93:e01823-18. doi:10.1128/JVI.01823-18 30429344 PMC6340017

[32] Marchitto L, Richard J, Prévost J, Tauzin A, Yang D, Chiu T-J, Chen H-C, Díaz-Salinas MA, Nayrac M, Benlarbi M, Beaudoin-Bussières G, Anand SP, Dionne K, Bélanger É, Chatterjee D, Medjahed H, Bourassa C, Tolbert WD, Hahn BH, Munro JB, Pazgier M, Smith AI, Finzi A. 2024. The combination of three CD4-induced antibodies targeting highly conserved Env regions with a small CD4-mimetic achieves potent ADCC activity. J Virol 98:e0101624. doi:10.1128/jvi.01016-24 39248460 PMC11495009

[33] Richard J, Nguyen DN, Tolbert WD, Gasser R, Ding S, Vézina D, Yu Gong S, Prévost J, Gendron-Lepage G, Medjahed H, Gottumukkala S, Finzi A, Pazgier M. 2021. Across functional boundaries: making nonneutralizing antibodies to neutralize HIV-1 and mediate fc-mediated effector killing of infected cells. mBio 12:e0140521. doi:10.1128/mBio.01405-21 34579568 PMC8546553

[34] Nikić I, Kang JH, Girona GE, Aramburu IV, Lemke EA. 2015. Labeling proteins on live mammalian cells using click chemistry. Nat Protoc 10:780–791. doi:10.1038/nprot.2015.045 25906116

[35] Richard J, Prévost J, Alsahafi N, Ding S, Finzi A. 2018. Impact of HIV-1 envelope conformation on ADCC responses. Trends Microbiol 26:253–265. doi:10.1016/j.tim.2017.10.007 29162391

[36] Decker JM, Bibollet-Ruche F, Wei X, Wang S, Levy DN, Wang W, Delaporte E, Peeters M, Derdeyn CA, Allen S, Hunter E, Saag MS, Hoxie JA, Hahn BH, Kwong PD, Robinson JE, Shaw GM. 2005. Antigenic conservation and immunogenicity of the HIV coreceptor binding site. J Exp Med 201:1407–1419. doi:10.1084/jem.20042510 15867093 PMC2213183

[37] Schuetz A, Deleage C, Sereti I, Rerknimitr R, Phanuphak N, Phuang-Ngern Y, Estes JD, Sandler NG, Sukhumvittaya S, Marovich M, et al.. 2014. Initiation of ART during early acute HIV infection preserves mucosal Th17 function and reverses HIV-related immune activation. PLoS Pathog 10:e1004543. doi:10.1371/journal.ppat.1004543 25503054 PMC4263756

[38] Finzi A, Xiang S-H, Pacheco B, Wang L, Haight J, Kassa A, Danek B, Pancera M, Kwong PD, Sodroski J. 2010. Topological layers in the HIV-1 gp120 inner domain regulate gp41 interaction and CD4-triggered conformational transitions. Mol Cell 37:656–667. doi:10.1016/j.molcel.2010.02.012 20227370 PMC2854584

[39] Mukherjee S, Dowd KA, Manhart CJ, Ledgerwood JE, Durbin AP, Whitehead SS, Pierson TC. 2014. Mechanism and significance of cell type-dependent neutralization of flaviviruses. J Virol 88:7210–7220. doi:10.1128/JVI.03690-13 24741083 PMC4054442

[40] Platt EJ, Wehrly K, Kuhmann SE, Chesebro B, Kabat D. 1998. Effects of CCR5 and CD4 cell surface concentrations on infections by macrophagetropic isolates of human immunodeficiency virus type 1. J Virol 72:2855–2864. doi:10.1128/JVI.72.4.2855-2864.1998 9525605 PMC109730

[41] Jain A, Govindan R, Berkman AR, Luban J, Díaz-Salinas MA, Durham ND, Munro JB. 2023. Regulation of ebola GP conformation and membrane binding by the chemical environment of the late endosome. PLoS Pathog 19:e1011848. doi:10.1371/journal.ppat.1011848 38055723 PMC10727438

[42] Díaz-Salinas MA, Li Q, Ejemel M, Yurkovetskiy L, Luban J, Shen K, Wang Y, Munro JB. 2022. Conformational dynamics and allosteric modulation of the SARS-CoV-2 spike. Elife 11:e75433. doi:10.7554/eLife.75433 35323111 PMC8963877

[43] Díaz-Salinas MA, Jain A, Durham ND, Munro JB. 2024. Single-molecule imaging reveals allosteric stimulation of SARS-CoV-2 spike receptor binding domain by host sialic acid. Sci Adv 10:eadk4920. doi:10.1126/sciadv.adk4920 39018397 PMC466946

[44] Das DK, Govindan R, Nikić-Spiegel I, Krammer F, Lemke EA, Munro JB. 2018. Direct visualization of the conformational dynamics of single influenza hemagglutinin trimers. Cell 174:926–937. doi:10.1016/j.cell.2018.05.050 29961575 PMC6086748

[45] Das DK, Bulow U, Diehl WE, Durham ND, Senjobe F, Chandran K, Luban J, Munro JB. 2020. Conformational changes in the Ebola virus membrane fusion machine induced by pH, Ca2+, and receptor binding. PLoS Biol 18:e3000626. doi:10.1371/journal.pbio.3000626 32040508 PMC7034923

[46] Blakemore RJ, Burnett C, Swanson C, Kharytonchyk S, Telesnitsky A, Munro JB. 2021. Stability and conformation of the dimeric HIV-1 genomic RNA 5’UTR. Biophys J 120:4874–4890. doi:10.1016/j.bpj.2021.09.017 34529947 PMC8595565

[47] Juette MF, Terry DS, Wasserman MR, Altman RB, Zhou Z, Zhao H, Blanchard SC. 2016. Single-molecule imaging of non-equilibrium molecular ensembles on the millisecond timescale. Nat Methods 13:341–344. doi:10.1038/nmeth.3769 26878382 PMC4814340

[48] Qin F, Auerbach A, Sachs F. 2000. A direct optimization approach to hidden Markov modeling for single channel kinetics. Biophys J 79:1915–1927. doi:10.1016/S0006-3495(00)76441-1 11023897 PMC1301083

[49] Huang J, Kang BH, Ishida E, Zhou T, Griesman T, Sheng Z, Wu F, Doria-Rose NA, Zhang B, McKee K, et al.. 2016. Identification of a CD4-binding-site antibody to HIV that evolved near-pan neutralization breadth. Immunity 45:1108–1121. doi:10.1016/j.immuni.2016.10.027 27851912 PMC5770152

