# Supplementary material for: Conformational dynamics of the HIV-1 envelope glycoprotein from CRF01_AE is associated with susceptibility to antibody-dependent cellular cytotoxicity
Source: J Virol. 2025 Dec 9;100(1):e01667-25. doi: 10.1128/jvi.01667-25 (PMC12817925; doi:10.1128/jvi.01667-25)
Supplement: Figure S1 — Plasma binding and ADCC responses. [file jvi.01667-25-s0001.pdf]

SUPPLEMENTAL MATERIAL

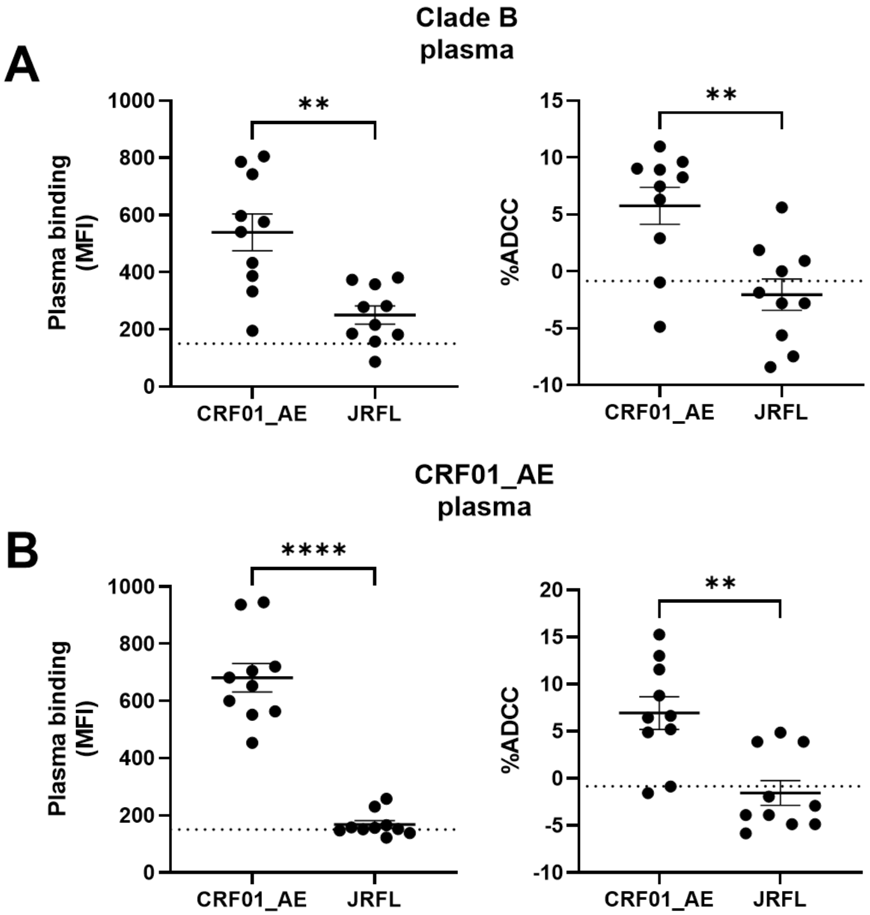

Fig S1. Plasma binding and ADCC responses against CRF01_AE (strain:703357) and Subtype B (JRFL) using plasma from (**A**) North American (Clade B) or (**B**) Thailand (CRF01_AE). Dotted lines represent the limit of detection calculated using 5 plasmas from uninfected individuals.
